# Complications and risk factors of percutaneous endoscopic transforaminal discectomy in the treatment of lumbar spinal stenosis

**DOI:** 10.1186/s12891-021-04940-z

**Published:** 2021-12-15

**Authors:** Ning Fan, Shuo Yuan, Peng Du, Qichao Wu, Tianyi Wang, Aobo Wang, Jian Li, Xiaochuan Kong, Wenyi Zhu, Lei Zang

**Affiliations:** Department of Orthopedics, Beijing Chaoyang Hospital, Capital Medical University, Beijing, China

**Keywords:** Percutaneous endoscopic transforaminal discectomy, Lumbar spinal stenosis, Complications, Risk factors

## Abstract

**Background:**

With the advancements in surgical methods, optical designs, and surgical instruments, percutaneous endoscopic transforaminal discectomy (PETD) has become an effective and minimally invasive procedure to treat lumbar spinal stenosis (LSS) in recent years. Few studies have focused on the complications associated with the treatment of LSS using percutaneous endoscopic lumbar discectomy (PELD). This study aimed to summarize the complications of PETD and identify the associated risk factors.

**Methods:**

Complications in a total of 738 consecutive LSS patients who underwent single-level PETD were retrospectively recorded and analyzed between January 2016 and July 2020. In addition, a matched case-control study was designed, and according to the date of operation, the control group was matched with patients without complications, with a matching ratio of 1:3. Demographic parameters included age, sex, BMI, smoking and drinking status, comorbidity, and surgical level. The radiological parameters included grade of surgical-level disc degeneration, number of degenerative lumbar discs, grade of lumbar spinal stenosis, degenerative lumbar scoliosis, lumbar lordosis, disc angle, and disc height index. Univariate analysis was performed using independent samples t-test and chi-squared test.

**Results:**

The incidence of different types of complications was 9.76% (72/738). The complications and occurrence rates were as follows: recurrence of LSS (rLSS), 2.30% (17/738); persistent lumbosacral or lower extremity pain, 3.79% (28/738); dural tear, 1.90% (14/738); incomplete decompression, 0.81% (6/738); surgical site infection, 0.41% (3/738); epidural hematoma, 0.27% (2/738); and intraoperative posterior neck pain, 0.27% (2/738). Univariate analysis demonstrated that age, the grade of surgical-level disc degeneration (*P* < 0.001) and the number of disc degeneration levels (*P* = 0.004) were significantly related to the complications.

**Conclusion:**

Complications in the treatment of LSS using PELD included rLSS, persistent pain of the lumbosacral or lower extremity, dural tear, incomplete decompression, surgical site infection, epidural hematoma, and intraoperative posterior neck pain. In addition, old age, severe grade of surgical-level disc degeneration and more disc degeneration levels significantly increased the incidence of complications.

## Background

Percutaneous endoscopic transforaminal discectomy (PETD), which is a percutaneous endoscopic lumbar discectomy (PELD), has become a routinely performed minimally invasive spinal procedure for the treatment of lumbar disc herniation (LDH) [1, 2]. However, with the advancements in surgical methods, optical designs, and surgical instruments, PETD has become the treatment option for lumbar spinal stenosis (LSS) in recent years [3–7]. PETD can not only remove disc herniation but also hyperplastic facet joints, hypertrophic ligamentum flavum, and osteophytes, which can increase the scale of the foramen, lateral recess, and even the central canal. According to a few studies, PETD showed clinical outcomes comparable to the conventional open decompression and fusion surgery, with advantages of lesser tissue trauma, faster rehabilitation, and lower intraoperative blood loss, risk of degeneration of adjacent segments and cost of postoperative care [8–10]. However, surgery-related complications have become more common and inevitable because of the widespread use of PETD, which makes patients dissatisfied with the postoperative efficacy.

The disadvantages of surgical techniques are the complications associated with the procedures. PETD has a sheer learning curve for beginners [1]. The appropriate working channel, which was established based on preoperative imaging and intraoperative fluoroscopy in PETD, is prone to complications, such as exiting nerve root and blood vessel injuries. In addition, PETD has difficulty in dealing with cases of L5/S1 because of the obstruction of the iliac crest [11]. Previous studies have reported a variety of complications, such as recurrence of LDH (rLDH), incomplete decompression, nerve root injury, dural tear, visceral injury, nerve root induced hyperalgesia or burning-like nerve root pain, postoperative dysesthesia, intraoperative seizure, posterior neck pain, surgical site infection, and instrument breakage, in the treatment of LDH using PELD [1, 2, 12–14]. In addition, these complications are associated with multiple factors, including rLDH-related risk factors, such as obesity, old age, Modic changes, low grade of surgical-level disc degeneration, high disc height index (DHI), large sagittal range of motion, and severe grade of adjacent-level disc degeneration [15–18].

However, only few studies have focused on the complications in PELD-based LSS treatment. Although the clinical manifestations of LSS are similar to those of LDH, there are significant differences in their pathogenesis, such as the factors compressing the nerve root, which makes the occurrence of surgery-related complications markedly different in both cases. In addition, a better understanding of the complications in PETD-based LSS treatment will help us accurately recognize the surgical indications and reduce the incidence of complications. Therefore, we conducted a retrospective study to summarize the complications of PETD in the treatment of LSS and identify the demographic and radiological risk factors of complications.

## Methods

### Patient selection

In the present study, 738 consecutive patients with unilateral lateral recess or foraminal stenosis who underwent single-level PETD were retrospectively evaluated in our department between January 2016 and July 2020. Imaging studies, including preoperative radiography, computed tomography (CT), and magnetic resonance imaging (MRI), were performed in all patients. The inclusion criteria were as follows: i) diagnosis of unilateral lateral recess or foraminal stenosis based on physical examination, clinical symptoms, and imaging studies; ii) symptoms with no relief after at least 3 months of conservative treatment. The exclusion criteria were as follows: i) symptoms caused only by LDH; ii) instability at the responsible level or more than grade-I spondylolisthesis; iii) more than one surgical level; iv) patients followed up for less than 1 year; v) history of lumbar surgery; and vi) concomitant conditions affecting the lumbar spine (fracture, tuberculosis, and tumor).

All operations were performed by a senior surgeon with an experience of more than 100 PELD procedures. All patients were administered local anesthesia. In patients with multilevel LSS, the responsible level was identified preoperatively through local blocking. The follow-up methods included outpatient services and telephone call surveys. For patients with severe symptoms post operation, MRI was performed and assessed for the presence of complications. In addition, a matched case—control design was used to identify the demographic and radiological risk factors. The case and control group comprised of patients with and without complications, respectively. The groups were matched according to the date of operation, and the matching ratio was 1:3. The investigation was approved by the institutional review board of the hospital, and the subjects provided informed consent prior to participation. All methods were carried out in accordance with the Declaration of Helsinki.

### Surgical methods

PETD was performed under local anesthesia, and patients could communicate with the surgeon during the entire operation, which prevented intraoperative nerve root damage. Patients were placed in prone position on a radiolucent table, and the operating and entry points were guided by fluoroscopy. A posterior midline paraspinal incision, which is normally 10–14 cm from the midline, was done by the surgeon for transforaminal approach. The guide wire was inserted into the superior articular process (SAP) of the targeted segment through a puncture needle under fluoroscopic guidance, and the surgical approach was progressively expanded to 8 mm using a hollow tapered cannula. A trepan was inserted into the cannula to perform the foraminoplasty. Next, the working cannula and endoscope were inserted into the incision. Next, the position of the working channel was confirmed using C-arm fluoroscopy. The hypertrophic ligamentum flavum and ventral elements of the SAP were removed using a rongeur; when necessary, trephine was used to further enlarge the foramen. The disc protrusion was completely resected using a rongeur, for ventral decompression. Following removal of the disc protrusion, ventral facets of the SAP and ligamentum flavum, the traversing nerve root and dural sac were exposed with adequate mobility and good pulse, indicating complete decompression. Finally, adequate irrigation and hemostatic treatment were performed, and the surgical wounds were sutured.

### Data collection and assessment

Demographic parameters, such as age, sex, BMI, smoking and drinking status, comorbidity, and surgical level, were collected. Radiological parameters, such as grade of surgical-level disc degeneration, number of degenerative lumbar discs, grade of lumbar spinal stenosis, degenerative lumbar scoliosis, lumbar lordosis, disc angle, and DHI, were compared between the case and control groups. Two trained orthopedic surgeons performed all the measurements using DICOM (version 3.1) viewer software (Neusoft PACS/RIS), and mean of the measurements was calculated. In addition, recurrence of LSS (rLSS) was defined as the relapse of LSS at the same level and on the same side in a patient with a pain-free interval of minimum one month after surgery [15].

The grade of surgical-level disc degeneration was assessed on T2-weighted sagittal sequences, according to the Pfirrmann criteria [19]. The number of degenerative lumbar discs was defined as the degenerations with Pfirrmann grade III or higher. The grade of lumbar spinal stenosis was based on the morphology of the dural sac on MRI, according to the criteria of Schizas et al. [20]. Degenerative lumbar scoliosis is a three-dimensional spinal deformity with a Cobb angle greater than 10 degrees [21, 22]. Lumbar lordosis was defined as the angle between the superior endplates of L1 and S1 [23]. According to the research results of Akeda et al. [24], DHI was calculated as [(Ha + Hp) / (Ds + Di)] x 100, where disc height measurements based on X-ray were as follows: Ha, anterior disc height; Hp, posterior disc height; Ds, superior disc depth; Di, inferior disc depth. Disc angle was the angle between the lower endplate of upper vertebra and the upper endplate of lower vertebra [25].

### Statistical analyses

All statistical analyses were performed using SPSS (version 23.0; IBM, USA). For continuous variables, data are presented as mean ± standard deviation. Univariate analysis was performed using independent samples t-test and chi-squared test for clinical and radiological parameters. Statistical significance was set at *p* < 0.05.

## Results

### Patient’s demographic characteristics

A total of 738 patients who underwent single-level PETD were enrolled in this study with a minimum follow-up period of one year. The follow-up period ranged from 12 to 60 months (21.4 ± 8.6 months). Seventy-two patients (39 men and 33 women with a mean age of 66.43 years (range, 47–89)) were diagnosed with different types of complications. A total of 216 patients were selected as matched controls from the remaining 766 patients without complications. The incidence of complications was 9.76% (72/738).

**Fig. 1:**
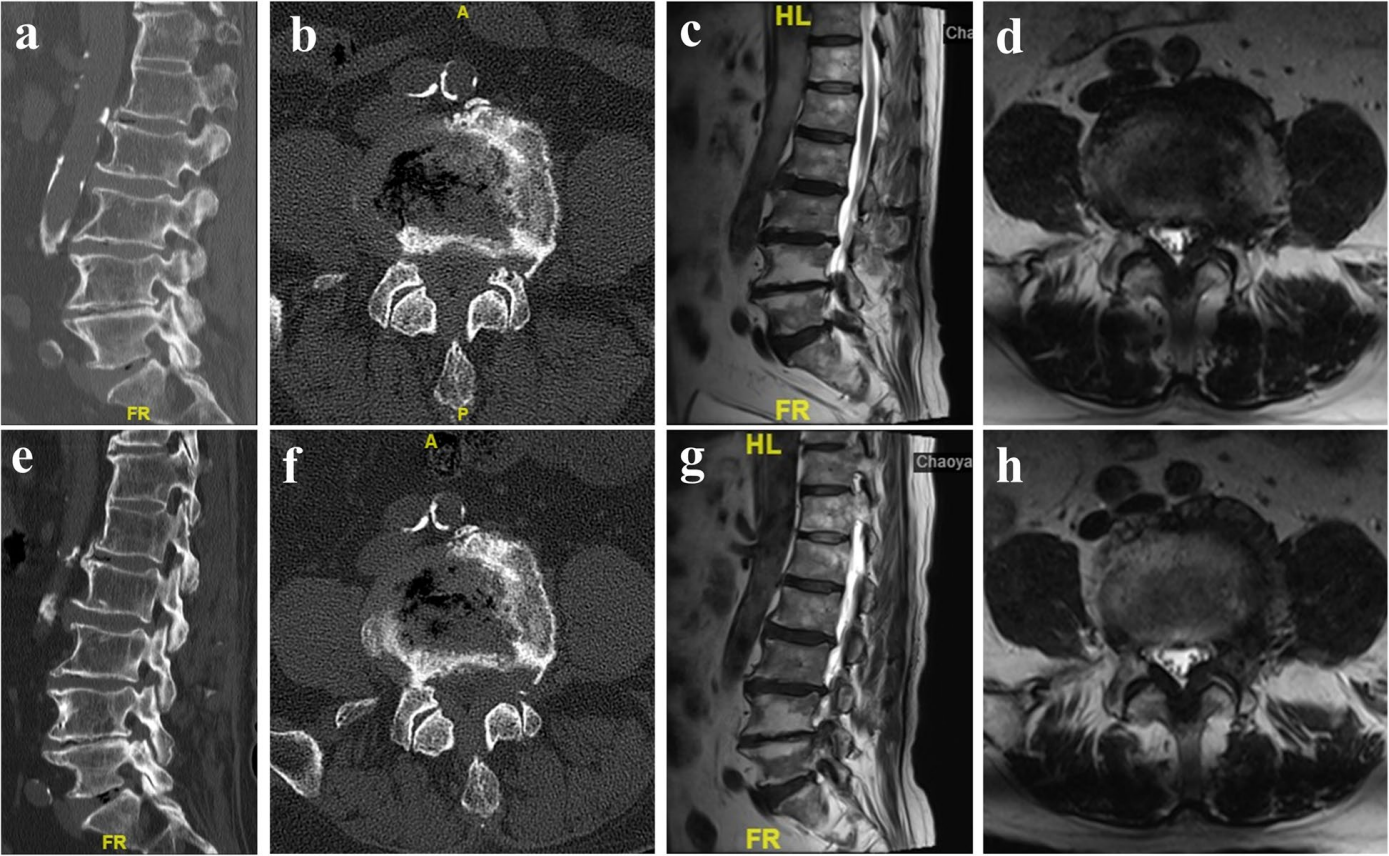
Case illustration of rLSS. An 83-year-old male patient underwent PETD for left-sided lateral recess stenosis at L4/5 level. Sagittal (**a**) and axial (**b**) CT showing left-sided hyperplastic facet joints and osteophyte at L4/5 level; sagittal (**c**) and axial (**d**) MRI showing left-sided disc herniation and hypertrophic ligamentum flavum; postoperative sagittal (**e**) and axial (**f**) CT demonstrating left-sided hyperplastic facet joints and osteophyte are removed; postoperative sagittal (**g**) and axial (**h**) MRI demonstrating the recurrence of LSS 6 months after PETD

**Fig. 2:**
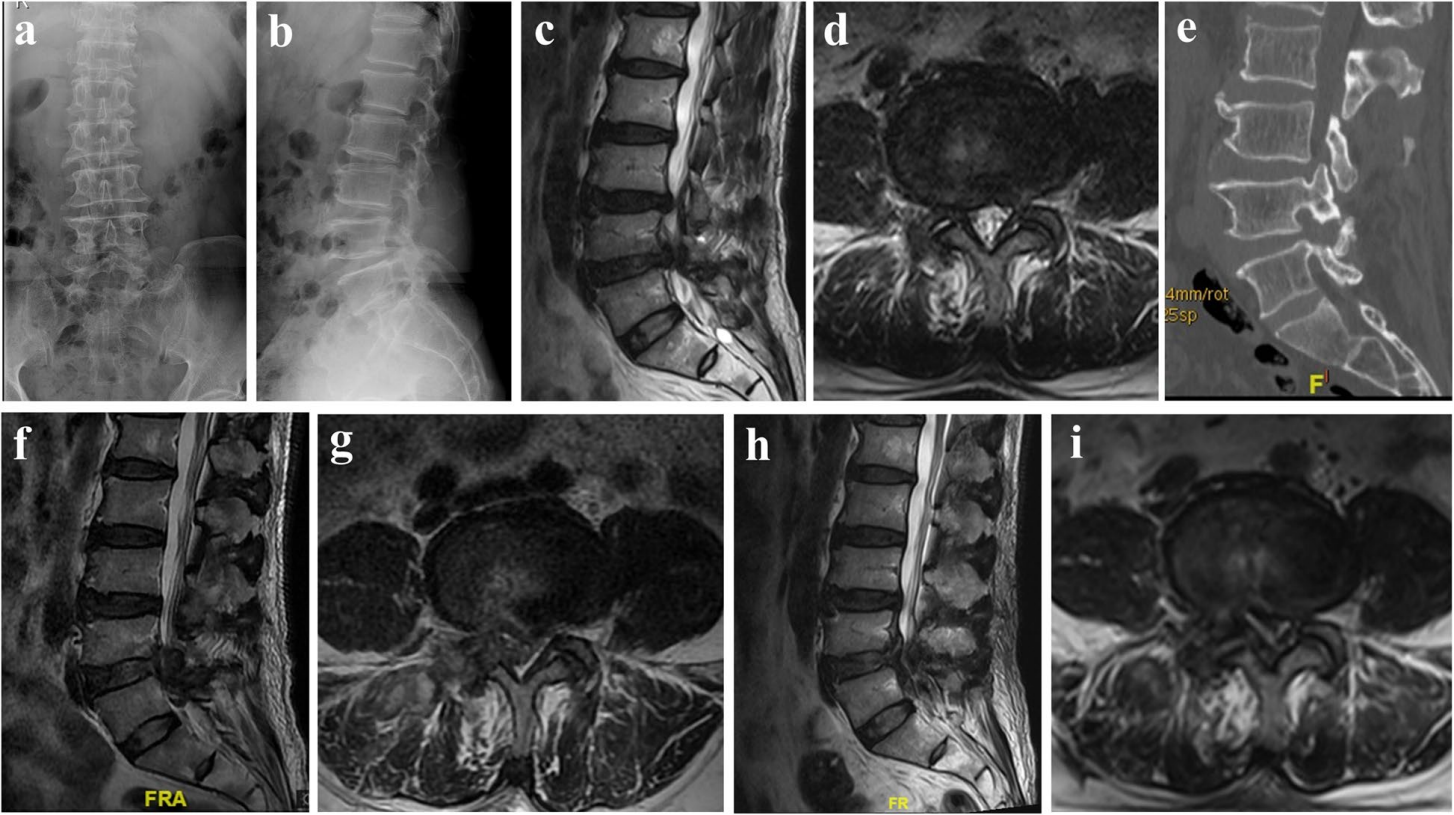
Case illustration of epidural hematoma. A 64-year-old male patient underwent PETD for right-sided lateral recess stenosis at L4/5 level. Posterior-anterior (AP) X-ray (**a**), lateral X-ray (**b**), sagittal (**c**), and axial (**d**) MRI showing right-sided lateral recess stenosis at L4/5 level; postoperative sagittal (**e**) CT showing foraminoplasty; postoperative sagittal (**f**) and axial (**g**) MRI demonstrating an epidural hematoma; postoperative sagittal (**h**) and axial (**i**) MRI after the second PETD demonstrating disappearance of epidural hematoma

The complications and occurrence rates were as follows: rLSS, 2.30% (17/738) (Fig. 1); persistent lumbosacral or lower extremity pain, 3.79% (28/738); dural tear, 1.90% (14/738); incomplete decompression, 0.81% (6/738); surgical site infection, 0.41% (3/738); epidural hematoma, 0.27% (2/738) (Fig. 2); intraoperative posterior neck pain, 0.27% (2/738). Of 72 patients with complications, 36 were treated conservatively, 16 were treated with PETD, 9 were treated with posterior lumbar interbody fusion, 1 was treated with endoscopic lumbar interbody fusion, and 10 were treated with local blocking (Table 1).

**Table 1:** The complications and treatments of 72 patients

Complications	No. of Patients	Occurrence rates (%)	Treatment
rLSS	17	2.30	9 patients were treated with PETD, 1 with endoscopic lumbar interbody fusion, 7 with PLIF.
Persistent lumbosacral or lower extremity pain	28	3.79	18 patients were treated conservatively, 10 with local blocking.
Dural tear	14	1.90	Intraoperative herniation of rootlets in 6 patients, intraoperative entrapment of rootlets in 2, and cauda equina was pulled out in 1. All of the patients were treated conservatively.
Incomplete decompression	6	0.81	3 patients were treated with PETD, 2 with PLIF; 1 patient refused revision and treated conservatively.
Surgical site infection	3	0.41	2 patients were treated with intervertebral washing by PETD and drainage, 1 with non-operation.
Epidural hematoma	2	0.27	Hematoma clearance by PETD.
Intraoperative posterior neck pain	2	0.27	Termination of the operation.

*rLSS* Recurrence of lumbar spinal stenosis, *PETD* Percutaneous endoscopic transforaminal discectomy, *PLIF* Posterior lumbar interbody fusion

The results of the univariate analyses of demographic characteristics are shown in Table 2. Except age (*p* = 0.007), there were no significant differences in sex (*p* = 0.838), BMI (*p* = 0.119), smoking status (*p* = 0.385), drinking status (*p* = 0.833), comorbidities (*p* > 0.05), and surgical level (*p* = 0.478) between cases and controls. Similarly, the results of the univariate analyses of radiological parameters are shown in Table 3. Univariate analysis demonstrated that the grade of surgical-level disc degeneration (*p* < 0.001) and number of disc degeneration levels (*p* = 0.004) were significantly related to the complications.

**Table 2:** Univariate analyses of demographic characteristics

Variable	Cases (n = 72)	Controls (n = 216)	*P*
Age (years)	66.43 ± 12.05	62.45 ± 10.16	0.007^*^
Sex (male/female), n	39/33	114/102	0.838
Body mass index (kg/m^2^)	26.04 ± 3.33	25.28 ± 3.61	0.119
Smoking status (yes), n (%)	15 (20.83)	56 (25.93)	0.385
Drinking status (yes), n (%)	9 (12.50)	25 (11.57)	0.833
Comorbidities			
Hypertension (yes), n (%)	44 (61.11)	105 (48.61)	0.066
Diabetes (yes), n (%)	17 (23.61)	64 (29.63)	0.325
Heart problem (yes), n (%)	13 (18.06)	31 (14.35)	0.449
Cerebrovascular disease (yes), n (%)	9 (12.50)	34 (15.74)	0.919
Surgical level, n (%)			0.478
L2/L3, L3/L4	3 (4.17)	17 (7.87)	
L4/L5	55 (76.39)	152 (70.37)	
L5/S1	14 (19.44)	47 (21.76)	

Asterisks indicate statistical significance (*p* < 0.05)

**Table 3:** Univariate analyses of radiological parameters

Variable	Cases (n = 72)	Controls (n = 216)	*P*
Grade of surgical-level disc degeneration, n (%)			0.000^*^
Grade I, II, III	2 (2.78)	62 (28.70)	
Grade IV	42 (58.33)	119 (55.09)	
Grade V	28 (38.89)	35 (16.21)	
Number of disc degeneration levels, n (%)			0.004^*^
One and two	24 (33.33)	118 (54.63)	
Three	26 (36.11)	62 (28.70)	
Four, five and six	22 (30.56)	36 (16.67)	
Grade of lumbar spinal stenosis, n (%)			0.540
A and B	41 (56.94)	125 (57.87)	
C	20 (27.78)	68 (31.48)	
D	11 (15.28)	23 (10.65)	
Degenerative lumbar scoliosis (yes), n (%)	16 (21.74)	28 (15.58)	0.059
Lumbar lordosis, (°)	36.18 ± 15.22	39.10 ± 13.77	0.131
Disc angle, (°)	9.18 ± 8.23	8.83 ± 4.42	0.650
Disc height index	0.247 ± 0.055	0.260 ± 0.052	0.059

Asterisks indicate statistical significance (*p* < 0.05)

## Discussion

Many previous studies have focused on the complications of PELD-based treatment of LDH [1, 2, 15]. However, few studies have reported complications and risks involved in the treatment of LSS with PELD. In the present study, we conducted a retrospective analysis of 738 patients with unilateral lateral recess or foraminal stenosis who had undergone PETD and found that 72 patients had surgery-related complications. The incidence rate of complications was 9.76%, which included rLSS, persistent lumbosacral or lower extremity pain, dural tear, incomplete decompression, surgical site infection, epidural hematoma, and intraoperative posterior neck pain. Accurate diagnosis and timely treatment play a significant role in the postoperative recovery of patients with complications. In addition, a matched case–control study was conducted and univariate analysis was used to identify the demographic and radiological risk factors that contributed to complications. According to our results, we found that old age, severe grade of surgical-level disc degeneration and more disc degeneration levels were significantly correlated with the incidence of complications.

rLSS is one of the major reasons for dissatisfaction among patients treated with PETD procedure. However, only few studies have reported the incidence of rLSS after PETD. In the present study, we defined rLSS as relapse of LSS of the same surgical level and on the same side in patients who had experienced a pain-free interval after surgery, and excluded contralateral LSS, which is different from the definition of rLDH [15]. For patients with bilateral stenosis, PETD is performed on one side of LSS, while contralateral LSS requires another PETD surgery, which obviously is different from rLSS. In this study, the incidence of rLSS was 2.30%, which was lower than that of rLDH after PELD (3.1–9.33%) [15–17]. Unlike conventional open decompression and fusion surgery, PETD only partially removed the factors causing LSS. Therefore, we found that rLSS is mainly caused by rLDH in the early follow-up, while rLSS can also be caused by hyperplastic facet joints, hypertrophic ligamentum flavum, and osteophytes in the long-term follow-up. If the compression factor of rLSS is mainly rLDH, PETD can be performed again. However, a higher incidence of rLDH still exists after PETD [26, 27]. In this study, 9 patients with rLSS underwent PETD, among whom 1 patient was re-diagnosed with rLDH after surgery and underwent PETD again. Furthermore, if the compression factors of rLSS are hyperplastic facet joints, hypertrophic ligamentum flavum, and osteophytes, conventional open or endoscopic decompression and fusion surgery is preferred.

Dural tear is one of the most common intraoperative complications of open and endoscopic spine surgery, and it is commonly caused by damage to the dura mater by surgical instruments and adhesion in the spinal canal [28]. In previous studies, the overall incidence of dural tear was 0–8.6% in endoscopic spine surgery, and the incidence was much higher in cases with LSS than in those with LDH [28, 29]. In our study, the incidence of dural tear was 1.90%. In this study, there were 14 patients with dural tears, 11 of which occurred at the nerve root sleeve. Since PETD mainly deals with unilateral lateral recess and foraminal stenosis, the most common location for dural tear is the nerve root sleeve, rather than the dural sac. In addition, 6, 2 and 1 patient suffered from intraoperative herniation of rootlets, intraoperative entrapment of rootlets, and a pulled-out cauda equina nerve, respectively. None of the patients with dural tear repaired the complication intraoperatively; they were treated conservatively, such as in the horizontal position for a period of time. None of the 13 patients experienced radiating pain or neurological defects. However, 3 days after surgery, 1 patient complained of intractable radiating pain in the lower extremities, which aggravated on changing position. Delayed herniation of a neural element from the dural tear was suspected, and the symptoms were relieved after immobilization in bed for a few days. Till now, there has been no consensus if dural tear requires repair [30, 31]. Furthermore, suturing is commonly not possible because of the limited working space for endoscopic procedures [32]. Thus, we believe that dural tears occurring during PETD are small lacerations that often heal spontaneously. However, open revision is required to suture the thecal sac and prevent further complications in patients with giant laceration [1, 30].

Previous studies have reported that the incidence of incomplete removal of herniated discs in patients with LDH treated by PETD was 1.4–2.8% [12, 33]. The location, type, and size of the herniated discs were closely related to their incomplete removal. Thus, detailed planning of the puncture route is important for complete removal. Similarly, incomplete decompression in LSS refers to the incomplete removal of herniated discs, hyperplastic facet joints, hypertrophic ligamentum flavum, and osteophytes. In this study, 6 patients had incomplete decompression. There were 3 patients with incomplete removal of herniated discs, among whom 2 patients underwent PETD again and 1 patient refused a surgical treatment. One patient had a nerve root compression by a bone mass that came from the foraminoplasty, which was removed during repeat PETD. The remaining 2 patients underwent conventional PLIF due to both anterior and posterior compression factors. We believe that for patients with incomplete decompression, the compression factors should be identified first, and then, surgical treatment (PETD or open lumbar interbody fusion) should be actively performed. Incomplete decompression needs to be differentiated from persistent lumbosacral or lower extremity pain, which can be differentiated using postoperative CT and MRI. Although the two had similar clinical manifestations, the treatments were completely different. In our study, a total of 28 patients were definitively diagnosed with persistent lumbosacral or lower extremity pain on postoperative CT and MRI. Of the 28 patients, 18 were treated conservatively, and 10 were treated with local blocking. We suggest that persistent lumbosacral pain may be associated with excessive intraoperative foraminoplasty, while persistent lower extremity pain may be associated with intraoperative nerve root injury and postoperative nerve root edema.

In addition, there are a few rare complications, such as surgical site infection, intraoperative posterior neck pain, and epidural hematoma. The low trauma associated with PELD makes surgical site infection uncommon [34]. However, pyogenic spondylodiscitis is a catastrophic complication that causes serious spinal nerve infection [1]. In our study, there were 3 patients with surgical site infection. Two of them were infected in the vertebral space of the operated segment, and treatments included intervertebral washing and drainage by PETD; intravenous antibiotics were administered postoperatively. The other patient who was infected during the surgical incision was only administered intravenous antibiotics. All three patients were cured without sequelae. The clinical manifestations of intraoperative neck pain occurred in 2 patients, among whom 1 patient was relieved after termination of surgery. The other patient subsequently developed loss of consciousness and respiratory cardiac arrest. The operation was immediately terminated and emergency treatment was provided. This patient later regained consciousness, and no intraoperative seizures or postoperative sequelae appeared. We believe that the occurrence of posterior neck pain is correlated with an increase in cervical epidural pressure, which may be caused by long operation time, fast lavage speed, and dural tear. When epidural pressure continues to increase, the patient may experience headaches, seizures, and even respiratory and heartbeat arrest [35, 36]. Therefore, surgery should be stopped when posterior neck pain occurs in patients. Two patients showed symptomatic postoperative epidural hematomas, as well as significant numbness and muscle weakness in the lower extremities after PETD. We diagnosed epidural hematoma using lumbar MRI and immediately performed hematoma cleaning using PETD. The symptoms of nerve root compression were relieved after the second surgery.

Although PETD can achieve good surgical efficacy in the treatment of LSS, its surgical indications remain controversial [9, 37]. Our study showed that old age, severe grade of surgical-level disc degeneration and more disc degeneration levels increased the incidence of complications, which indicates that patients with such conditions should be cautious about PETD. The previous studies showed that the prevalence of rLDH after PELD was significantly higher in older patients, which is consistent with our conclusion [17, 38]. We believe that older discs are characterized by increased degeneration, and severe disc degeneration reduces the height of the foramina and aggravates the hyperplasia of facet joints, thus reducing the operating space of PETD and increasing the difficulty of surgery. In addition, the proliferation of pannus around the degenerated disc leads to intraoperative bleeding, which results in an unclear surgical field. The above reasons significantly increase the incidence of complications. In addition, we assume that severe disc degeneration at the adjacent level may indirectly increase stress load at the surgical level due to biomechanical conduction [15], which promotes rLDH. Therefore, the more disc degeneration levels, the higher the incidence of rLDH.

This study has several limitations. First, this was a retrospective single-center study, and the case group had a relatively small number of cases. The occurrence of a selection bias cannot be eliminated. Second, only those with severe symptoms of LSS, rather than all patients after primary surgery, underwent postoperative MRI. Some patients did not undergo lumbar MRI, even though they had definite symptoms of rLSS during follow-up, which may have led to a true incidence of complications higher than 9.76%. Third, although our study found significant statistical differences in radiological parameters between the case and control groups, it did not adequately reflect the correlation between these radiological parameters and each type of complication. Therefore, we will further analyze the risk factors related to certain complications in subsequent studies. Finally, the follow-up period was relatively short, which also had an impact on the incidence of complications. Although our study had these limitations, we believe that they do not substantially detract from the conclusions of this study.

## Conclusions

PETD is an effective and minimally invasive method for dealing with LSS. However, there are a variety of complications associated with it. In our study, the incidence of complications was 9.76%, and these complications included rLSS, persistent lumbosacral or lower extremity pain, dural tear, incomplete decompression, surgical site infection, epidural hematoma, and intraoperative posterior neck pain. In addition, it was found that old age, severe grade of surgical-level disc degeneration and more disc degeneration levels significantly increased the incidence of complications.

## Data Availability

The datasets used and/or analysed during the current study are available from the corresponding author on reasonable request.

## Abbreviations

PETD: Percutaneous endoscopic transforaminal discectomy
PELD: Percutaneous endoscopic lumbar discectomy
LDH: Lumbar disc herniation
LSS: Lumbar spinal stenosis
rLDH: Recurrence of lumbar disc herniation
DHI: Disc height index
CT: Computed tomography
MRI: Magnetic resonance imaging
SAP: Superior articular process
rLSS: Recurrence of LSS
PLIF: Posterior lumbar interbody fusion
